# Chrysin Attenuates VCAM-1 Expression and Monocyte Adhesion in Lipopolysaccharide-Stimulated Brain Endothelial Cells by Preventing NF-κB Signaling

**DOI:** 10.3390/ijms18071424

**Published:** 2017-07-03

**Authors:** Bo Kyung Lee, Won Jae Lee, Yi-Sook Jung

**Affiliations:** 1 College of Pharmacy, Ajou University, Suwon 16499, Korea; pfiffer@ajou.ac.kr (B.K.L.); wjlee@ilyang.co.kr (W.J.L.); 2 Research Institute of Pharmaceutical Sciences and Technology, Ajou University, Suwon 16499, Korea

**Keywords:** chrysin, neuroinflammation, blood-brain barrier, vascular cell adhesion molecule-1 (VCAM-1), lipopolysaccharide, monocyte adhesion, brain endothelial cell

## Abstract

Adhesion of leukocytes to endothelial cells plays an important role in neuroinflammation. Therefore, suppression of the expression of adhesion molecules in brain endothelial cells may inhibit neuroinflammation. Chrysin (5,7-dihydroxyflavone) is a flavonoid component of propolis, blue passion flowers, and fruits. In the present study, we examined the effects of chrysin on lipopolysaccharide (LPS)-induced expression of vascular cell adhesion molecule-1 (VCAM-1) in mouse cerebral vascular endothelial (bEnd.3) cells. In bEnd.3 cells, LPS increased mRNA expression of VCAM-1 in a time-dependent manner, and chrysin significantly decreased LPS-induced mRNA expression of VCAM-1. Chrysin also reduced VCAM-1 protein expression in a concentration-dependent manner. Furthermore, chrysin blocked adhesion of monocytes to bEnd.3 cells exposed to LPS. Nuclear factor-κB (NF-κB), p38 mitogen-activated protein kinase (MAPK), and c-Jun N-terminal kinase, which are all activated by LPS, were significantly inhibited by chrysin. These results indicate that chrysin inhibits the expression of VCAM-1 in brain endothelial cells by inhibiting NF-κB translocation and MAPK signaling, resulting in the attenuation of leukocyte adhesion to endothelial cells. The anti-inflammatory effects of chrysin suggest a possible therapeutic application of this agent to neurodegenerative diseases, such as multiple sclerosis, septic encephalopathy, and allergic encephalomyelitis.

## 1. Introduction

Neuroinflammation, inflammation of the central nervous system (CNS), occurs in response to lesions caused by various conditions, including infection, traumatic brain injury, and autoimmunity [1]. Neuroinflammation is known to play a role in neurodegenerative diseases, such as multiple sclerosis (MS), Parkinson’s disease, and septic encephalopathy [2,3,4]. MS, specifically, is the most common neuroinflammatory autoimmune disease, and it is characterized by inflammation in the brain and spinal cord that damages the myelin sheath, thereby causing demyelination of neurons. Cerebral microvascular endothelial cells (CMECs), which form the blood-brain barrier (BBB), are essential for the maintenance of homeostasis and for restricting immune cell access to the CNS. CMECs play important roles in the initial stages of brain disorders, including MS pathogenesis, by upregulating cellular adhesion molecules such as intercellular adhesion molecule-1 (ICAM-1), vascular cell adhesion molecule-1 (VCAM-1), and E-selectin, which causes them to adhere to inflammatory cells and leads to migration of inflammatory cells into the brain [1,5]. Cellular adhesion molecules on the surfaces of cerebral endothelial cells enhance the local inflammatory response within cerebral vessels and the brain parenchyma by recruiting and inducing the transmigration of leukocytes [6,7,8]. ICAM-1 and VCAM-1, specifically, are constitutively expressed in CMECs [9]. Various stimuli such as lipopolysaccharide (LPS), a bacterial component that induces inflammation, upregulate ICAM-1 and VCAM-1, and changes in the expression of these molecules promote cerebral vascular inflammation, BBB disruption, and vasogenic edema [10].

Chrysin (5,7-dihydroxyflavone), a flavonoid component of propolis, blue passion flowers, and fruits, has been reported to have anti-inflammatory, anti-allergic, and anti-cancer effects [11,12,13]. In mouse microglial cells, chrysin has been shown to play a role as a suppressor of LPS-induced inflammation by inhibiting the release of nitric oxide (NO), tumor necrosis factor-α (TNF-α), and interleukin-1β (IL-1β). Chrysin has also shown inhibitory effects on neuroinflammation by repressing the expression of pro-inflammatory cytokines and upregulating astrocytic protein expression in hippocampal neurons [14]. Recently, chrysin has been reported to attenuate TNF-α-induced upregulation of ICAM-1 and E-selectin, but not VCAM-1, in aortic endothelial cells [15] and to inhibit TNF-α-induced upregulation of ICAM-1 through activator protein-1 (AP-1) and nuclear factor-κB (NF-κB) in respiratory epithelial cells [12]. However, information on the effects of chrysin on brain endothelial cells and their regulatory mechanisms is lacking. In the present study, the effects of chrysin on the expression of adhesion molecules in mouse brain endothelial cells (bEnd.3) were analyzed, and NF-κB-mediated signal transduction and mitogen-activated protein kinase (MAPK) expression were assessed in chrysin-treated bEnd.3 cells, to better understand the mechanisms underlying the beneficial effects of chrysin in neuroinflammatory diseases.

## 2. Results

### 2.1. Chrysin Inhibits LPS-Induced VCAM-1 mRNA Expression in bEnd.3 Cells

**Figure 1:**
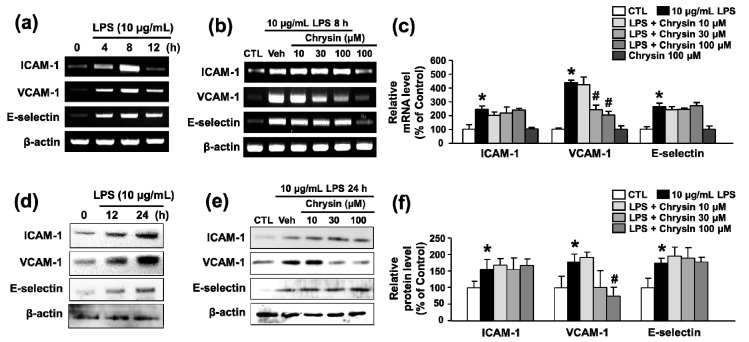
The effect of chrysin on LPS-induced adhesion molecule mRNA and protein expression in bEnd.3 cells. (**a**) RT-PCR of adhesion molecules after 10 μg/mL LPS treatment. bEnd.3 cells were treated with 10 μg/mL LPS for 4, 8, or 12 h; (**b**) RT-PCR of adhesion molecules after LPS and chrysin treatment. Cells were pretreated with chrysin (10–100 μM) for 30 min and co-stimulated with 10 μg/mL LPS for 8 h; (**c**) the levels of ICAM-1, VCAM-1, and E-selectin mRNA were evaluated by RT-PCR and quantified by densitometry; (**d**) Western blotting of adhesion molecules after 10 μg/mL LPS treatment. bEnd.3 cells were treated with 10 μg/mL LPS for 12 or 24 h; (**e**) Western blotting of adhesion molecules after LPS and chrysin treatment. Cell were pretreated with chrysin (10–100 μM) for 30 min and co-stimulated with 10 μg/mL LPS for 24 h; and (**f**) the levels of ICAM-1, VCAM-1, and E-selectin protein were evaluated by Western blotting and quantified by densitometry. β-actin was used for normalization. Mean ± SEM, * *p* < 0.05 vs. control and # *p* < 0.05 vs. 10 μg/mL LPS alone, *n* = 3.

To determine whether chrysin inhibits inflammation, we performed RT-PCR and Western blotting to evaluate the mRNA and protein expression of adhesion molecules. We used 10 μg/mL LPS, a commonly used concentration for inducing inflammation in endothelial cells [16]. Treatment of bEnd.3 cells with 10 μg/mL LPS significantly increased the levels of ICAM-1, E-selectin, VCAM-1 mRNA, and protein in a time-dependent manner (4–12 h and 12–24 h, respectively) (Figure 1a,d). Chrysin treatment inhibited the mRNA expression of VCAM-1 in LPS-induced bEnd.3 cells in a concentration-dependent manner (10–100 μM, 435.8 ± 19.2% to 201.4 ± 28.6% at 100 μM), but not ICAM-1 and E-selectin (243.6 ± 25.2% to 239.0 ± 12.9% and 263.9 ± 26.3% to 271.3 ± 21.5% at 100 μM, respectively) (Figure 1b,c). LPS-induced protein expression of VCAM-1 also inhibited by chrysin (Figure 1e,f). This finding indicates that chrysin inhibits the expression of VCAM-1 following stimulation of endothelial cells by LPS.

### 2.2. Chrysin Inhibits LPS-Induced VCAM-1 Protein Expression and Monocyte Attachment in bEnd.3 Cells

**Figure 2:**
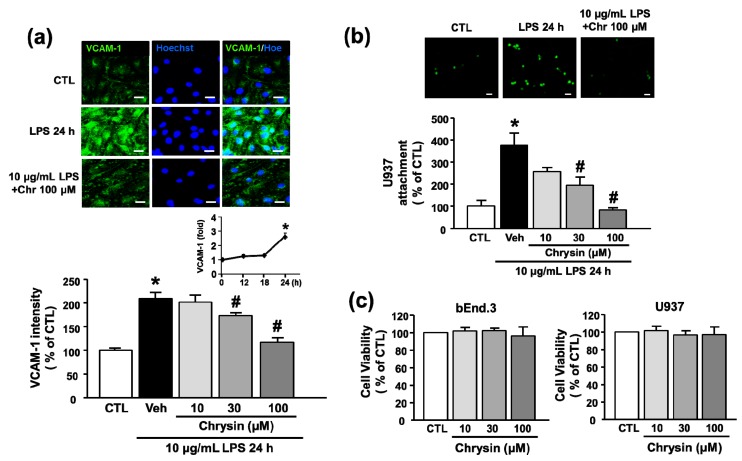
The effect of chrysin on LPS-induced VCAM-1 protein expression and U937 cells attachment to bEnd.3 cells. (**a**) Immunocytochemistry after LPS and chrysin treatment. Inset: bEnd.3 cells were exposed to 10 μg/mL LPS for the indicated times (0–24 h). Cells were pretreated with chrysin (10–100 μM) for 30 min and co-stimulated with 10 μg/mL LPS for 24 h. VCAM-1 (green) was detected by immunofluorescence using anti-VCAM-1 antibody. Nuclei were visualized by Hoechst staining (blue). Representative images and quantitative results are shown in the top and bottom panels, respectively. Scale bar, 20 μm; (**b**) after treatment with 10 μg/mL LPS for 24 h in the presence or absence of chrysin, bEnd.3 cells were incubated with U937 monocytes (green) for 2 h. Representative images and quantitative results are shown in the top and bottom panels, respectively. Scale bar: 40 μm; and (**c**) the effect of chrysin alone on cell viability in bEnd.3 and U937 cells. After the treatment with various concentrations of chrysin for 24 h, the cytotoxicity was determined by MTT assay. Mean ± SEM, * *p* < 0.05 vs. control and # *p* < 0.05 vs. vehicle, *n* = 3.

To verify the inhibitory effects of chrysin on LPS-induced VCAM-1 protein expression, we performed immunocytochemistry. Treatment of bEnd.3 cells with 10 μg/mL LPS for 24 h significantly increased the levels of VCAM-1 protein (100.0 ± 5.0% to 209.2 ± 13.0%), and VCAM-1 expression was decreased by treatment with 30 and 100 μM chrysin (172.6 ± 6.9% and 117.8 ± 9.8%) (Figure 2a). One of the earliest events in inflammation is binding of monocytes to the endothelium and infiltration of monocytes through these cells. Therefore, monocyte-endothelial interactions are important to the development of inflammatory diseases [17]. To determine whether chrysin inhibits inflammation at an early stage, we performed a cell–cell adhesion assay. Treatment of bEnd.3 cells with 10 μg/mL LPS significantly increased the number of U937 cells attached to activated endothelial cells at 24 h (100.0 ± 27.0% to 374.5 ± 55.4%). Chrysin treatment inhibited the adhesion of monocytes to LPS-stimulated bEnd.3 cells in a concentration-dependent manner (Figure 2b). In particular, 100 μM chrysin decreased the attachment of U937 cells to that of the control level (83.9 ± 9.4%). This finding indicates that chrysin inhibits the attachment of monocytes to endothelial cells by blocking the stimulation of endothelial cells with LPS. Cell viability was measured to demonstrate that the reduction of U937 attachment induced by chrysin was not due to cytotoxicity. As shown in Figure 1c, the 10–100 μM chrysin did not show any cytotoxic effect in both bEnd.3 and U937 cells.

### 2.3. Chrysin Inhibits LPS-Induced VCAM-1 Expression by Blocking NF-κB Translocation in bEnd.3 Cells

**Figure 3:**
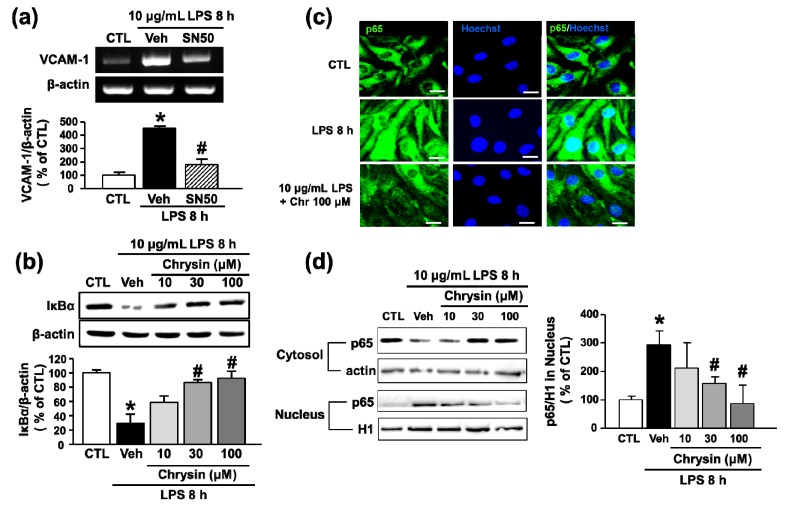
The effect of chrysin on LPS-induced translocation of NF-κB in bEnd.3 cells. (**a**) Cells were pretreated with SN50 for 30 min and co-stimulated with LPS (10 μg/mL) for 8 h. The levels of VCAM-1 mRNA were evaluated by RT-PCR and quantified by densitometry; (**b**) cells were pretreated with chrysin (10–100 μM) for 30 min and then stimulated with LPS (10 μg/mL) for 8 h. Whole cell and nuclear extracts were prepared and analyzed by Western blot analysis with anti-IκBα antibody. The levels of protein were normalized with β-actin; (**c**) cells were pretreated with chrysin (10–100 μM) for 30 min and then co-stimulated with LPS (10 μg/mL) for 8 h. NF-κB p65 (green) was detected by immunofluorescence using anti-p65 antibody. Nuclei were visualized by Hoechst staining (blue). Scale bar: 20 μm; and (**d**) Western blots of p65 subunit of NF-κB in nucleus and cytosolic fraction of bEnd.3 cells treated with LPS (10 μg/mL) and chrysin for 8 h. Actin was used as internal standard of cytosol fraction and histone 1 (H1) of the nuclear fraction. * *p* < 0.05 vs. control without LPS and # *p* < 0.05 vs. vehicle without SN50, *n* = 3.

NF-κB signaling is associated with the onset of various inflammatory autoimmune diseases such as leukemia, inflammatory bowel disease, arthritis, sepsis, asthma, and MS [18]. Recently, chrysin was reported to attenuate inflammatory mediator-induced adhesion molecule expression via transcription factor NF-κB signaling in respiratory epithelial cells [12]. Therefore, to examine whether NF-κB played a role in LPS-induced VCAM-1 expression and whether chrysin inhibited LPS-induced VCAM-1 expression through NF-κB signaling in bEnd.3 cells, we used the NF-κB inhibitor SN50. The results showed that SN50 inhibited VCAM-1 mRNA after 8 h of LPS treatment (452.2 ± 16.7% to 180.2 ± 42.5%) (Figure 3a). To determine whether treatment with chrysin would inhibit NF-κB signaling, IκBα degradation and NF-κB translocation to the nucleus were investigated. After pretreatment with chrysin, bEnd.3 cells were exposed to LPS for 8 h. Chrysin significantly attenuated LPS-induced IκBα degradation (29.7 ± 16.8% to 94.6 ± 11.3% at 100 μM) (Figure 3b) and LPS-induced NF-κB p65 subunit translocation from the cytosolic to the nuclear fraction of bEnd.3 cells (Figure 3c,d). This finding indicates that chrysin blocks LPS-induced upregulation of VCAM-1 through NF-κB signaling in brain endothelial cells.

### 2.4. Chrysin Attenuates LPS-Induced VCAM-1 Expression by Inhibiting p38MAPK and JNK Phosphorylation in bEnd.3 Cells 

**Figure 4:**
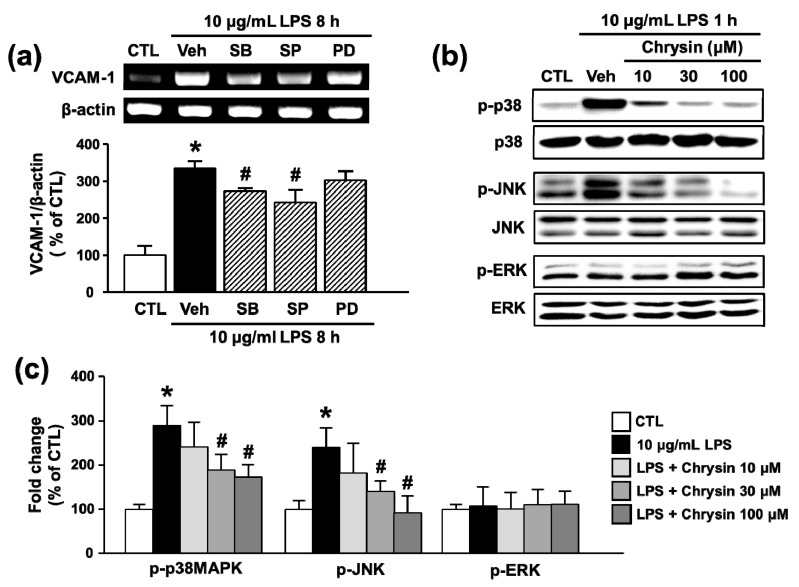
Effect of MAPK on LPS-induced VCAM-1 expression and of chrysin on p38 and JNK activation in bEnd.3 cells. (**a**) Cells were pretreated with SB202190 (SB, 10 μM), SP600125 (SP, 10 μM), and PD98059 (PD, 10 μM) for 30 min and then co-stimulated with LPS (10 μM) for 8 h. The levels of VCAM-1 mRNA were evaluated by RT-PCR and quantified by densitometry; and (**b**,**c**) cells were pretreated with chrysin (10–100 μM) for 30 min and then co-stimulated with LPS (10 μg/mL) for 1 h. Whole cell extracts were prepared and analyzed by Western blot with anti-phospho p38, anti-phospho JNK, p38 MAPK antibody, or JNK antibody. * *p* < 0.05 vs. control and # *p* < 0.05 vs. vehicle, *n* = 3.

In this study, we examined the relationship between MAPKs and LPS-induced VCAM-1 expression in bEnd.3 cells. The p38MAPK and JNK inhibitors SB202190 and SP600125, respectively, significantly decreased LPS-induced upregulation of VCAM-1 mRNA (333.3 ± 20.7% to 272.0 ± 8.3% and 241.7 ± 34.7%, respectively), but MEK inhibitor PD98059, used to inhibit extracellular signal-regulated kinase (ERK), did not reduce VCAM-1 mRNA levels (302.7 ± 23.7%) (Figure 4a). These results suggested that p38MAPK and JNK are involved in LPS-induced VCAM-1 expression. In addition, we investigated whether chrysin inhibited LPS-induced activation of MAPKs such as p38MAPK, JNK, and ERK by phosphorylation in bEnd.3 cells. As shown in Figure 4b,c, phosphorylation of p38MAPK and JNK was decreased by chrysin in a dose-dependent manner (p38MAPK: 289.8 ± 45.3% to 173.2 ± 28.5% and JNK: 240.2 ± 44.4% to 92.0 ± 39.0% at 100 μM); however, ERK phosphorylation was not affected (107.4 ± 43.5% to 112.1 ± 29.5% at 100 μM). These results show that chrysin suppresses LPS-induced VCAM-1 expression by inhibiting p38MAPK and JNK signaling.

### 2.5. Chrysin Attenuates LPS-Induced VCAM-1 Expression by Inhibiting p38MAPK and JNK Phosphorylation in bEnd.3 Cells 

**Figure 5:**
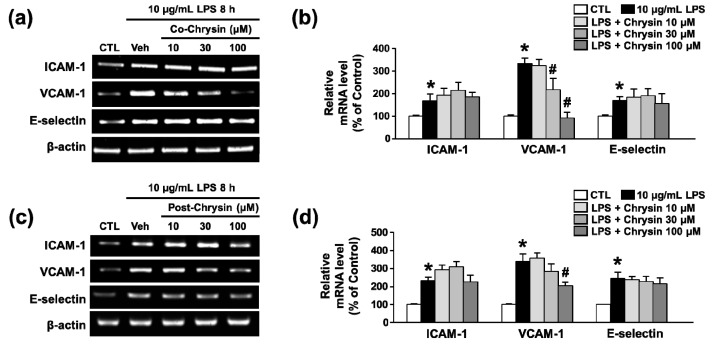
The therapeutic effect of chrysin on LPS-induced adhesion molecule mRNA expression in bEnd.3 cells. (**a**) Cells were co-treated with chrysin (10–100 μM) and 10 μg/mL LPS for 8 h; (**b**) the levels of ICAM-1, VCAM-1, and E-selectin mRNA were evaluated by RT-PCR and quantified by densitometry; (**c**) cell were post-treated with chrysin (10–100 μM) at 4 h after administration of 10 μg/mL LPS; and (**d**) the levels of ICAM-1, VCAM-1, and E-selectin mRNA were evaluated by RT-PCR and quantified by densitometry. β-actin was used for normalization. Mean ± SEM, * *p* < 0.05 vs. control and # *p* < 0.05 vs. 10 μg/mL LPS alone, *n* = 3.

To provide possibility of the therapeutic effects of chrysin in an established disease condition, we examined the protective potential of chrysin through the co-treatment and 4 h post-treatment of chrysin with 10 μg/mL LPS in bEnd.3 cells. Co-treatment with chrysin inhibited the mRNA expression of VCAM-1 in LPS-induced bEnd.3 cells in a concentration-dependent manner, but not ICAM-1 and E-selectin (Figure 5a,b). Similarly to the results of co-treatment with chrysin and LPS, 4 h post-treatment with chrysin also inhibited only VCAM-1 expression (Figure 5c,d). These findings indicate that chrysin plays an anti-inflammatory role by arresting the progression of LPS-induced inflammation during disease conditions.

## 3. Discussion

In the present study, we examined whether chrysin alleviates the inflammatory response to LPS in CMECs. Treatment with chrysin after LPS stimulation decreased the levels of VCAM-1, NF-κB, JNK, and p38MAPK, which are all known as inflammatory mediators. Indeed, administration of chrysin reduced monocyte attachment to CMECs. This suggests that chrysin limited adhesion of monocytes to endothelial cells, probably by decreasing VCAM-1 expression in CMECs.

Numerous studies have shown that VCAM-1 plays a crucial role in the migration of immune cells to the CNS across the BBB [19,20]. Adhesion of inflammatory cells to the vascular endothelium represents an early stage in cell migration into the brain parenchyma. First, leukocytes are captured and “slow rolling” occurs to hold leukocytes near the endothelial cells, which leads to chemokine-induced leukocyte activation and the expression of other inflammatory factors on the surface of endothelial cells [21]. Next, cell adhesion molecules, such as VCAM-1, ICAM-1, and selectins, enhance tight adhesion of leukocytes. In particular, α4β1 integrin expressed on lymphocytes and monocytes binds to VCAM-1 and causes trans-endothelial migration of cells [22,23,24,25]. Through interactions between α4β1 integrin on lymphocytes and VCAM-1 on endothelial cells, the leukocytes attach to the blood vessel wall and then begin the process of diapedesis, or “walking through,” the blood vessel wall [26].

MS is a fatal autoimmune disease characterized by brain inflammation, which damages brain infiltration by self-reactive immune cells. In MS, VCAM-1 has a significant effect on the migration of immune cells. In addition, α4β1 integrin is known to be important in MS pathogenesis. In 1992, Yednock first published evidence that α4β1 integrin plays a critical role in the induction of lymphocytes to the inflamed brain, and that antibodies to α4β1 integrin or its ligand VCAM-1 blocked MS pathogenesis and inhibited the development of experimental autoimmune encephalomyelitis (EAE), the most commonly used experimental model for MS [5,27]. Many other cell adhesion molecules (e.g., ICAM-1 and selectin) that were expected to play a role in the binding of lymphocytes to the inflamed brain (EAE blood vessels) were ineffective. Antibodies to L-selectin did not affect binding to inflammatory blood vessels in rat EAE [28]. Although expression of ICAM-1 was evident in EAE blood vessels, anti-β2 integrin binding to ICAM-1 had no effect on lymphocyte binding [29]. This may relate to differences in the relative levels of VCAM-1 and ICAM-1 in brain endothelial cells. A previous study suggested that VCAM-1 and ICAM-1 contribute differently to leukocyte action during brain inflammation, because the expression of adhesion molecules differs depending on tissue type [30]. The levels of VCAM-1 in brain endothelial cells were found to be higher than those in other tissues, in both inflamed and non-inflamed brains [31]; however, ICAM-1 was expressed at a uniform level in many tissues, including the brain. Therefore, the role of VCAM-1 may be more important than that of ICAM-1 in inflammatory brain diseases such as MS.

Recently, studies have shown that LPS induces upregulation of cell adhesion molecules including ICAM-1, VCAM-1, and E-selectin in brain endothelial cells, indicating the mechanism underlying inflammation [32,33]. Consistent with these reports, our study showed that LPS increased the levels of cell adhesion molecules such as ICAM-1, VCAM-1, and E-selectin in CMECs. We found that although chrysin administration notably suppressed the expression of VCAM-1, it did not suppress the expression of ICAM-1 and E-selectin. Chrysin treatment effectively attenuated the adhesion of monocytes to CMECs, indicating that the possible anti-attachment effects of chrysin are specifically associated with the inhibition of VCAM-1 in CMECs. The anti-attachment properties of chrysin were confirmed by its effective inhibition of pro-inflammatory NF-κB activity. NF-κB is an important transcription factor that upregulates the expression of adhesion molecules in CMECs, which is a major mechanism underlying inflammation and autoimmune diseases [34]. In previous studies, the NF-κB signaling pathway was shown to regulate VCAM-1 expression in CMECs [6,35] [6,35] and to be activated by various inflammatory stimuli such as LPS. In addition, the p38MAPK and JNK pathways were found to mediate inflammatory signaling stimulated by LPS, cytokines, and stress factors such as oxidation and other shocks, whereas activation of p44/42MAPK mediates cell proliferation in response to growth factors and mitogens [36,37,38]. In this study, VCAM-1 expression was partially suppressed by inhibition of NF-κB, p38MAPK, or JNK (SN50, SB202190, or SP600125) respectively, but not p44/42MAPK, in CMECs exposed to LPS. These findings suggest that the induction of VCAM-1 expression by LPS may depend on activation of NF-κB and p38MAPK/JNK signaling. NF-κB and p38MAPK/JNK signaling may play in the same signaling on VCAM-1 activity, or may be synchronized by parallel working in both. However, our results, together with those presented by Kempe et al. [39] indicate that VCAM-1 expression requires synergistic activation of NF-κB and p38MAPK/JNK, and that both pathways are essential for this expression. Phosphorylation of p38MAPK/JNK has been reported to activate transcription factors like Elk-1, AP-1, and CREB [39]. Additionally, crosstalk between the transcription factors NF-κB and AP-1 in inflammatory cells induced by LPS has been investigated [40]. In the present study, the expression of VCAM-1 might have enabled NF-κB, in cooperation with p38MAPK/JNK, to evoke a transcriptional response in cells activated by LPS, and chrysin treatment might have suppressed VCAM-1 expression by inhibiting NF-κB and p38MAPK/JNK signaling. However, further analyses are needed to define the role that chrysin plays in orchestrating inflammation elicited by LPS in brain endothelial cells.

Natalizumab is a human monoclonal antibody against α4β1 integrin that blocks the migration of lymphocytes to disease sites. It has been approved by the FDA for the treatment of the autoimmune disease MS. However, within three months of approval, natalizumab was withdrawn from use because of reports of progressive multifocal leukoencephalopathy (PML) in two patients who received natalizumab in clinical trials. The most potent inducers of PML in response to natalizumab treatment are integrins, which are known to act as receptors for many viruses, including the JC virus that causes PML [41]. Despite the therapeutic effects of inhibiting immune cell adhesion and VCAM-1 expression in MS, these effects have severely limited the introduction of this drug into clinical use. Therefore, it is important to find a novel therapeutic agent for MS that selectively inhibits both immune cell adhesion and VCAM-1 expression. This study showed that chrysin inhibits the adhesion of inflammatory cells and selectively inhibits the expression of VCAM-1, thereby suggesting the potential for use of chrysin to effectively control neuroinflammatory diseases such as MS.

## 4. Materials and Methods

### 4.1. Cell Culture and Reagents

bEnd.3 cells, a mouse brain endothelial cell line, and U937, a human monocyte cell line, were purchased from American Type Culture Collection (ATCC) (Manassas, VA, USA). The bEnd.3 cells were maintained in Dulbecco’s modified Eagle’s medium (DMEM) (Gibco-BRL, Grand Island, NY, USA) with 10% fetal bovine serum (FBS, Gibco-BRL) and 1% penicillin streptomycin (PS, Invitrogen, Carlsbad, CA, USA). The U937 cells were maintained in Roswell Park Memorial Institute (RPMI) medium (Gibco-BRL) supplemented with 10% FBS and 1% PS. Cells were incubated in an atmosphere of 95% air and 5% CO_2_ at 37 °C. Cells were pretreated with chrysin or inhibitors for 30 min before exposure to LPS in DMEM with 1% FBS. Chrysin and inhibitors were dissolved in 1% dimethyl sulfoxide (DMSO), and LPS was dissolved in distilled water. The final concentration of DMSO was 0.1%, which was found to have no effect on cell viability. LPS and chrysin were both obtained from Sigma-Aldrich (St. Louis, MO, USA). High-glucose DMEM, FBS, PS, and trypsin-EDTA were obtained from Invitrogen. SN50, SP600125, SB202190, and PD98059 were obtained from Calbiochem (La Jolla, CA, USA).

### 4.2. Reverse-Transcription Polymerase Chain Reaction (RT-PCR)

Total RNAs were isolated using an easy-BLUE extraction kit (Intron Biotechnology, Seongnam, Korea). By measuring the 260/280 nm absorbance ratios, DNA concentration and the purity of samples were determined. cDNA was synthesized from total RNA (2 μg) by using random hexamers and Moloney murine leukemia virus (M-MLV) reverse transcriptase (ServLab, Seoul, Korea), and then was amplified by PCR using Taq DNA polymerase, dNTPs, and gene-specific primers. The primers used in this study were as follows: VCAM-1, 5′-CCC AAG GAT CCA GAG ATT CA-3′ (forward), 5′-TAA GGT GAG GGT GGC ATT TC-3′ (reverse); ICAM-1, 5′-GAA GGT GGT TCT TFT GAG CG-3′ (forward), 5′-GTC TGC TGA GAC CCC TCT TG-3′ (reverse); and E-selectin, 5′-AGC TAC CCA TGG AAC ACG AC-3′ (forward), 5′-TGC AAG CTA AAG CCC TCA TT-3′ (reverse). PCR products were separated on a 2% agarose gel and stained with EcoDye DNA staining solution (SolGent, Daejeon, Korea).

### 4.3. Preparation of Cytosolic or Nuclear Extracts

Cytosolic and nuclear extracts were obtained as described previously [35]. Cells were lysed in a buffer containing 50 mM Tris, 10 mM NaCl, 1 mM ethylenediaminetetraacetic acid (EDTA), 1 mM dithiothreitol (DTT), 1 μM leupeptin, 1 μM pepstatin, 1 μM aprotinin, and 1 μM phenylmethylsulfonyl fluoride (PMSF). After centrifugation at 18,000× *g* for 10 min at 4 °C, the nuclei were extracted from the pellets. The cytosolic fractions (supernatant) were separated, and the nuclei were extracted from the pellets at 4 °C in the same buffer as before, except with the addition of 0.4 M NaCl.

### 4.4. Western Blotting

Western blot analysis was performed as described previously [42]. Cells were washed with phosphate-buffered saline (PBS, Gibco-BRL) twice and lysed on ice in RIPA buffer (pH 7.4) comprising 150 mM NaCl, 1% NP-40, 0.5% Na-deoxycholate, 50 mM Tris-HCl (pH 7.4), 1 mM EDTA, and protease inhibitors (Sigma Aldrich, St. Louis, MO, USA). The cells were centrifuged at 20,000× *g* for 15 min at 4 °C, and cell debris was removed. The collected supernatants were boiled and electrophoresed on a 10% sodium dodecyl sulfate (SDS) polyacrylamide gel. To detect clear data, more than 30 μg of total protein from a cell lysate loaded. Proteins were electro-transferred to membranes and incubated with anti-ICAM-1, anti-VCAM-1, anti-E-selectin, anti-Iκ-Bα, anti-p65, anti-H1, anti-β-actin, anti-p44/42, anti-phospho p-44/42, anti-JNK, anti-phospho JNK, anti-p38, or anti-phospho p38 antibodies at 4 °C, followed by incubation with the appropriate horseradish peroxidase-conjugated secondary antibody for 2 h at 20 °C. Bands were detected using enhanced chemiluminescence reagent (Intron Biotechnology, Seongnam, Korea), and images were captured using an Image-Quant Las 4000 system (GE Healthcare, Madison, WI, USA). All antibodies were purchased from Cell Signaling (Beverly, MA, USA).

### 4.5. Immunocytochemistry

bEnd.3 cells grown on cover glasses were washed with 1× HEPES-buffered control salt solution (HCSS) and fixed in 4% paraformaldehyde (PFA) for 10 min after exposure to LPS and chrysin treatment. Cells were incubated with 3% bovine serum albumin (BSA)-HCSS blocking solution for 30 min at room temperature, with anti-VCAM-1 and anti-p65 (Santa Cruz Biotechnology Inc. Santa Cruz, CA, USA) antibodies overnight at 4 °C, and then with secondary antibody labeled with Alexa Fluor 488 (Molecular Probes, Eugene, OR, USA) for 2 h. Cell nuclei were stained with Hoechst 33258 (Molecular Probes) for 10 min, and all samples were observed under a Carl Zeiss confocal microscope (LSM 410; Carl Zeiss, Jena, Germany).

### 4.6. Adhesion Assay

An adhesion assay was performed as previously described [43]. bEnd.3 cells, grown in 24-well plates, were treated with LPS at 37 °C for 24 h after pretreatment with chrysin for 30 min and then washed twice with PBS. U937 cells were labeled for 30 min at 37 °C with 2 μM 5-chloromethylfluorescein diacetate (CMFDA, Molecular Probes), washed twice with PBS, and suspended in growth medium. Then, 2.5 × 10^5^ labeled cells were added to the bEnd.3 monolayer at a final volume of 500 μL and incubated in a CO_2_ incubator for 2 h at 37 °C on a block light. Non-adherent cells were removed from the plate by gentle washing with PBS, and the number of adherent cells was determined by measuring the fluorescence intensity under a fluorescence microscope (Carl Zeiss, Germany).

### 4.7. 3-[4,5-Dimethyl-Triazolyl-2]2,5-Diphenyl Tetrazolium Bromide (MTT) Assay

MTT assay for cell viability was performed as previously described [44]. Cells were seeded in 96-well plates at a density designed to reach 90% confluency at the time of assay. Cells were treated with various concentrations of chrysin in triplicate. After 24 h of chrysin treatment, MTT was added at 0.5 mg/mL finally, and the plate was incubated for 1 h at 37 °C. Cells having functional mitochondrial succinate dehydrogenase can convert MTT to formazan that generates a blue color when dissolved in dimethyl sulfoxide (DMSO). DMSO was added and the absorbance was read at 540 nm using a BioTek spectrophotometer (Winooski, VT, USA).

### 4.8. Statistical Analysis

Data are expressed as mean ± standard error of the mean of three separate determinations for each group. Numerical data were compared using Student’s *t*-test or one-way ANOVA post hoc test for unpaired observations between two groups. A *p*-value < 0.05 was considered significant.

## Abbreviations

VCAM-1	Vascular Cell Adhesion Molecule-1
LPS	Lipopolysaccharide
NF-κB	Nuclear Factor-κB
MAPK	Mitogen-Activated Protein Kinase
CMECs	Cerebral Microvascular Endothelial Cells
BBB	Blood-Brain Barrier
MS	Multiple Sclerosis
